# The Plant V-ATPase

**DOI:** 10.3389/fpls.2022.931777

**Published:** 2022-06-30

**Authors:** Thorsten Seidel

**Affiliations:** Dynamic Cell Imaging, Faculty of Biology, Bielefeld University, Bielefeld, Germany

**Keywords:** V-ATPase, proton pump, pH-homeostasis, Arabidopsis, glucose

## Abstract

V-ATPase is the dominant proton pump in plant cells. It contributes to cytosolic pH homeostasis and energizes transport processes across endomembranes of the secretory pathway. Its localization in the trans Golgi network/early endosomes is essential for vesicle transport, for instance for the delivery of cell wall components. Furthermore, it is crucial for response to abiotic and biotic stresses. The V-ATPase’s rather complex structure and multiple subunit isoforms enable high structural flexibility with respect to requirements for different organs, developmental stages, and organelles. This complexity further demands a sophisticated assembly machinery and transport routes in cells, a process that is still not fully understood. Regulation of V-ATPase is a target of phosphorylation and redox-modifications but also involves interactions with regulatory proteins like 14-3-3 proteins and the lipid environment. Regulation by reversible assembly, as reported for yeast and the mammalian enzyme, has not be proven in plants but seems to be absent in autotrophic cells. Addressing the regulation of V-ATPase is a promising approach to adjust its activity for improved stress resistance or higher crop yield.

## Introduction

**FIGURE 1:**
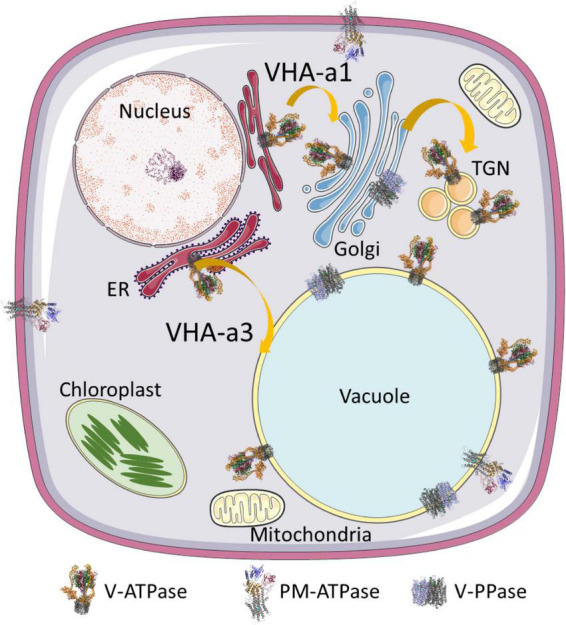
Localization of proton pumps. V-ATPase is present in all endomembranes of the secretory pathway, including ER membranes. V-PPases are localized in the vacuole and rarely in the Golgi, while PM-ATPases are present in the plasma membrane and, in some tissues, even in tonoplasts. Two transport routes exist for V-ATPases. Proton pumps destined for TGN/EE bear VHA-a1 and travel along the secretory pathway, while VHA-a3-bearing pumps are destined for the vacuole and take a direct route from the ER to the vacuole. The structure of V-PPase relies on pdb file 6afs and that of the plasma membrane on file 5 ksd (Croll and Andersen, 2016; Tsai et al., 2019). The structure of V-ATPase is based on pdb 3j9t (Zhao et al., 2015).

The secretory pathway provides membrane-bound proteins, luminal proteins, and lipids to cells. Furthermore, it is responsible for posttranslational modifications like glycosylation and serves as a sorting point for transport of the extracellular material regardless of its direction, endocytosis, or exocytosis. Organelles of the secretory pathway are characterized by a specific luminal pH, which is highest and close to the cytosolic pH of 7.2 at the ER (pH 7.1) and decreases with the following compartments, *cis*-Golgi (pH 6.8), *trans*-Golgi network (TGN, pH 6.3), multivesicular bodies (MVBs, pH 6.2), late endosomes (pH 5.3), and the vacuole (pH 5.2) (Grabe and Oster, 2001; Shen et al., 2013). The luminal pH ensures an environment of matching the required reaction conditions in organelles, and the proton gradient across endomembranes provides the required driving force for transport across membranes (Grabe et al., 2000). Proton-translocating ATPases of vacuolar type acidify the lumen of Golgi, TGN, late endosomes, and the vacuole as demonstrated by their concanamycin A-sensitivity. Treatment with the V-ATPase inhibitor concanamycin A results in increased pH of TGN and the vacuole, while the pH of ER, *cis*-Golgi, and MVBs is unaffected, but Golgi swelling has been observed in BY2-cells (Robinson et al., 2004; Shen et al., 2013). The function of V-ATPases in TGN and the vacuole has been intensively investigated in recent years. In the vacuole, at least two proton pumps exist, the vacuolar proton translocating pyrophosphatase (V-PPase) and V-ATPase (Figure 1). V-PPase is the dominant proton pump in early developmental stages of plants like fruits or organs, whereas V-ATPase takes over in later stages and becomes the dominant pump (Shiratake et al., 1997). Both proton pumps are required in tonoplasts during embryo development, and lack of V-PPase and V-ATPase results in altered vacuolar morphology and defects in auxin transport by dislocation of PIN1 in tonoplasts (Krebs et al., 2010; Jiang et al., 2020). However, V-PPases are capable of compensating for loss of active V-ATPases in vma1^–^ yeast cells lacking VHA-A (Perez-Castineira et al., 2011), whereas in Arabidopsis and rice lack of V-ATPase cannot be compensated by V-PPases because of limitations in subcellular distribution (Zhang et al., 2013; Kriegel et al., 2015). In later developmental stages, the major role of V-PPases seems to be the cytosolic pyrophosphate (PP_i_) scavenging to avoid PP_i_ toxicity by forming insoluble complexes with Mg^2+^ or Ca^2+^(Segami et al., 2018). V-PPases were also reported to function in a reversible manner, producing PP_i_ using the proton gradient of V-ATPase. Such conversion of the energy stored in the proton gradient may transform the energy back into a biochemically accessible form Scholz-Starke et al. (2019). In some specialized cell types, even P-type proton-translocating ATPases were found (Figure 1), for instance the pump AHA10 in the seed coat endothelium of *Arabidopsis thaliana* (Appelhagen et al., 2015). In petunia, two P-type ATPases, PH1 and PH5, function as a heteromeric pump in tonoplasts, and control the color of flowers (Verweij et al., 2008; Faraco et al., 2014). Interestingly, loss of V-ATPase function results in internalization of the plasma membrane proton pump Pma1p in yeast, although such compensation mechanism has not been observed in plants (Velivela and Kane, 2018).

In 1997, Matsuoka et al. (1997) reported that a non-vacuolar V-ATPase is required for sorting of vacuolar protein precursors. It turned out later that the V-ATPase in TGN/EE is essential for many processes in plants including salt tolerance, delivery of cell wall components, recycling of plasma membrane proteins, and compensated cell expansion, and that it contributes to vacuolar acidification so loss of vacuolar proton pumps can be counterbalanced (Bruex et al., 2008; Ferjani et al., 2013; Kriegel et al., 2015; Luo et al., 2015; Tsuyama et al., 2019). In the vacuole, V-ATPase energizes mainly transport processes, contributes to nitrate storage and zinc deposition, ensures resting calcium concentration in the cytosol (Krebs et al., 2010; Han et al., 2016), and plays a role in guard cell by regulating stomatal opening (Allen et al., 2000; Zhang et al., 2013). In contrast to the equal distribution of V-PPases in tonoplasts, V-ATPase was found in specific regions that might represent vacuolar microdomains and facilitate the interplay with secondary active transporters (Yoshida et al., 2013; Siek et al., 2016). Last but not least, V-ATPases were found in peribacteroid membranes and endosidin bodies, the latter containing the TGN-specific subunit VHA-a1 and seem to originate from endosomes by interference of endosidin 1 and endocytosis (Saalbach et al., 2002; Robert et al., 2008).

Due to its impact on cellular transport and regulation of luminal and cytosolic pH-homeostasis, V-ATPase is of different importance for different cell types. Especially, regions of cell elongation depend on active V-ATPases. Failure of V-ATPase activity in pollen results in cytoplasmic acidification and finally affects mitochondrial function by collapse of the membrane potential, which leads to retarded pollen growth in *Pyruspyrifolia* (Gao et al., 2015). Pollen-specific VHA-E isoforms are reported for higher plants, but they have no essential function in gametophyte development and are incapable of compensating for loss of other isoforms (Strompen et al., 2005; Dettmer et al., 2010).

V-ATPase might further be involved in homotypic vacuolar fusion as described for yeast V-ATPase, in particular its V_0_-sector, by forming stacks of proteolipid rings that form a fusion pore subsequently (Peters et al., 2001; Wilson et al., 2021). V-ATPases might also be involved in vacuolar-endosomal fusion (Wilson et al., 2021). Proteolipids are capable of forming a conductance pore, which supports the hypothesis of a function in membrane fusion (Couoh-Cardel et al., 2016). Other findings indicated the requirement of V-ATPase activity for vacuolar fusion and not direct involvement of the V_0_-structure (Coonrod et al., 2013). Vacuolar acidification by V-ATPases plays also a role for cellular aging in yeast (Stephan et al., 2013; Ghavidel et al., 2018); they act as a cellular timer of vacuoles and vacuoles increase by size and undergo more frequent contact with other organelles and, thus, have been denominated as hubs of cellular homeostasis (Aufschnaiter and Buttner, 2019).

## Composition of the Complex

Initially, structural analysis relied on X-ray analysis of isolated subunits and low resolution images by transmission electron microscopy. Thus, the assembly of the complex has been a hassle and resulted in well-meant but less precise superposition of electron micrographs and single pdb files. The structural analysis of V-ATPase has benefitted from the improvement of high resolution cryo-electron microscopy and enzyme reconstitution in nano-discs. In recent years, multiple structures of yeast and human V-ATPases of dissociated and fully assembled states and three different rotational states were published.

**FIGURE 2:**
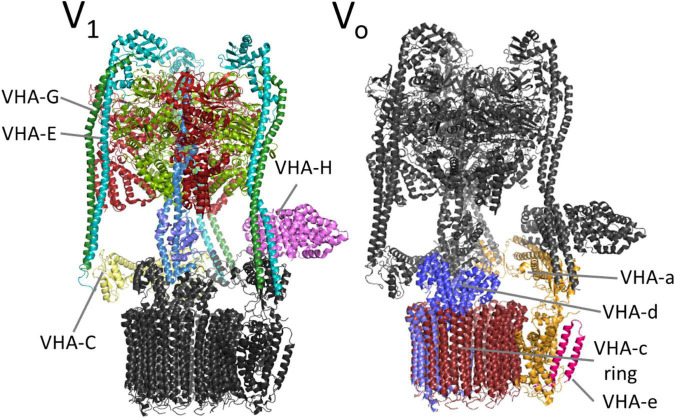
V-ATPase complex. V-ATPase comprises of the membrane integral subsector V_0_ and the membrane-associated subsector V_1_. V_0_ is responsible for proton transport and dominated by a ring composed of ten molecules of proteolipid VHA-c. A single proteolipid is highlighted in blue (VHA-c”). ATP hydrolysis takes place in V_1_, in particular in VHA-A, and drives the rotation of the central stalk by VHA-D and VHA-F. The peripheral subunits serve as a scaffold and anchor the complex to the membrane *via* VHA-a. The structure is based on pdb7tmr (Vasanthakumar et al., 2022).

**FIGURE 3:**
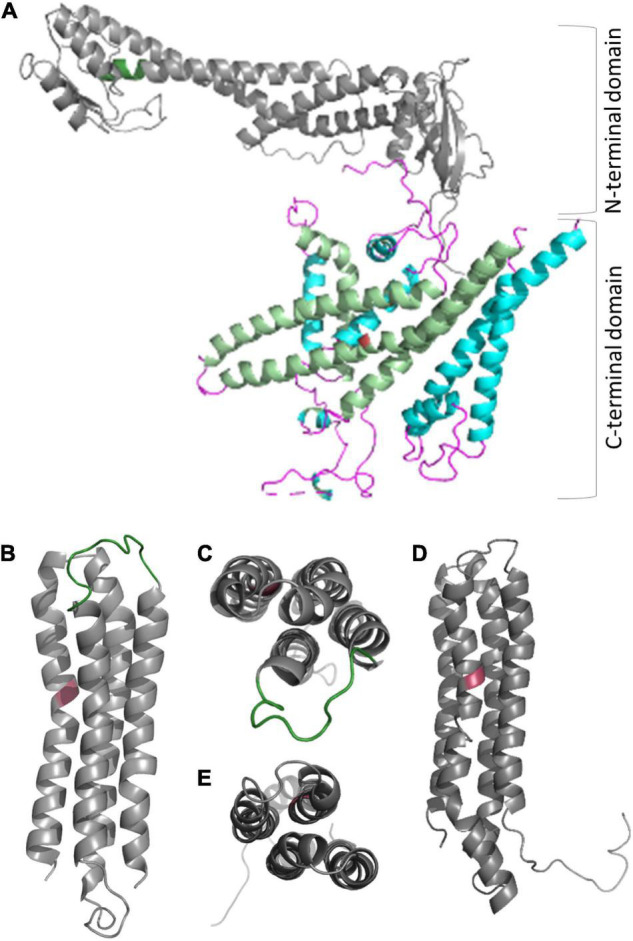
Structure of proteolipids VHA-c and VHA-c” and subunit VHA-a. **(A)** VHA-a has a bipartite structure of the cytosolic N-terminal domain and the C-terminal membrane integral domain. The N-terminal domain serves as part of stator and bears the TGN localization domain in the case of VHA-a1 (position is indicated by dark green color). Helices of the C-terminal domain form semi-channels for proton loading and unloading of VHA-c (light green helices), and the barrier charge surrounds a conserved arginine residue on helix seven (red colored). **(B–D)** The proteolipids consist of four transmembrane helices [refer to the view from the cytosolic side: **(C,E)**], and the protein binding site is a conserved glutamate residue (red color) located in the fourth helix [VHA-c; **(B)**] or the second helix [VHA-c”, **(D)**]. A cytosolic loop of VHA-c (green color) serves as a binding site for VHA-d. Structures were obtained with Phyre2 (Kelley et al., 2015), and a complete set of pdb files for *A. thaliana* VHA subunits is available as Supplementary Data.

The complex has a bipartite structure of more than 800 kDa, formed by the subcomplexes V_0_ and V_1_, which represent the proton transporter and the transport-driving ATPase, respectively (Figure 2). V_0_ consists of the subunits VHA-a, VHA-c, VHA-c”, VHA-d, and VHA-e, while V_1_ consists of subunits VHA-A to VHA-H (Sze et al., 2002). Both subsectors are dominated by central symmetric arrangement of subunits; for V_0_ it is a ring of the proteolipids VHA-c and VHA-c”, which bear conserved glutamic acid residues as proton-binding sites in a flexible transmembrane helix (Wang et al., 2004). Initially, the proteolipid ring was thought to consist of six proteolipids, but recent data from yeast and mammals demonstrated that ten proteolipids actually form the proteolipid ring (Roh et al., 2018, 2020; Abbas et al., 2020). The yeast or fungal-specific proteolipid VHA-c’ is not present in plants and other higher eukaryotes, although it is required for assembly of the complex in fungal cells (Sze et al., 2002; Chavez et al., 2006). Every proteolipid VHA-c has a molecular mass of 16 kDa and consists of four transmembrane helices, VHA-c” also has four helices, but it has a molecular mass of 18 kDa (Seidel et al., 2008). The proton binding glutamate residue is located in the fourth and second helix of VHA-c and VHA-c”, respectively (Figure 3; Wang et al., 2004). In Arabidopsis, three isoforms of VHA-c exist and are encoded by five genes. The isoforms VHA-c1, VHA-c3, and VHA-c5 are identical at the protein level. VHA-c” is encoded by two genes, resulting in the isoforms VHA-c”1 and 2. Both VHA-c” isoforms were identified in the ER and were absent in the vacuole in plants (Jaquinod et al., 2007; Seidel et al., 2008). In the ring structure, the distance between proton binding sites is constant except for rings bearing VHA-c”. The uneven spacing caused by VHA-c” has been suggested to enable auto-inhibition in yeast, fixing the proteolipid ring in a position with VHA-c”, which is close to transmembrane helices 7 and 8 of VHA-a in the inhibited state (Roh et al., 2018). However, VHA-c” has also been shown to be part of the binding pocket of VHA-d during V-ATPase assembly (Roh et al., 2018), so it might take over the function of VHA-c’ for complex assembly in higher eukaryotes. The proteolipid ring is flanked by the C-terminal domain of VHA-a, which forms two water-filled hemi-channels for cytosolic proton access to the proteolipid ring, and their final release into the organelle lumen and might further serve as a pH sensor that communicates the endosomal pH to the cytosolic side (Grabe et al., 2000; Marshansky, 2007; Roh et al., 2020). Based on the topology of the yeast VHA-a subunit Vph1p, VHA-a consists of eight transmembrane helices, and helices 7 and 8 form the cytoplasmic hemi-channel and helices 3, 4, and 7 the luminal one (Figure 3); both semi-channels are characterized by charged and polar amino acid residues (Toei et al., 2011; Vasanthakumar et al., 2019). VHA-a further carries a positive barrier charge with a conserved arginine residue (Arg 735 in yeast) in its center, which affects the pKa of proton-binding glutamate residues and supports the release of protons. The helix bearing this barrier charge shows dynamic flexibility, which might be required for proper channel gating and positioning of the barrier charge (Duarte et al., 2007; Vos et al., 2007). The N-terminal domain of VHA-a is hydrophilic and exposed to the cytosol. It serves as a membrane anchor for the V_1_ sector and interacts with the proteolipid ring in the absence of V_1_ (Roh et al., 2018). VHA-a isoforms define the subcellular localization of V-ATPase, differing between TGN/EE- and vacuolar isoenzymes in plants. This difference in localization has been observed in yeast first; here, the isoform Stv1p locates to the Golgi, and Vph1p was found in the vacuolar membrane (Manolson et al., 1994). Stv1p targeting to the Golgi relies on a WKY-motif, which interacts with phosphatidylinositol 4-phosphate (PI(4) in the Golgi (Finnigan et al., 2012; Vasanthakumar et al., 2019). Furthermore, a more negative surface charge of Vph1p results in higher proton concentration in the cytoplasmic hemi-channel and, hence, in higher activity than Stv1p (Vasanthakumar et al., 2019). In Arabidopsis, the isoforms VHA-a2 and VHA-a3 were identified in the vacuolar membrane, while VHA-a1 was found in the TGN (Dettmer et al., 2006). Lupanga et al. (2020) identified a plant-specific motif for TGN-targeting in VHA-a1 that is different from the WKY-motif. To avoid ion leakage, the 41-kDa subunit VHA-d resides on the cytosolic side of the ring, interacts with cytoplasmic loops of VHA-c, and blocks the central pore (Roh et al., 2018). The open proteolipid ring would form such a central pore with a conductance of 8.3 nS (Ouyang et al., 2008; Couoh-Cardel et al., 2016), and without VHA-d, V_0_ would be a passive proton channel (Ouyang et al., 2008). Early reports of VHA-d being part of the stator were not confirmed by recent structures (Thaker et al., 2008; Roh et al., 2020). In Arabidopsis, two isoforms, VHA-d1 and VHA-d2, exist and are encoded by two neighboring genes (Sze et al., 2002). Two isoforms are also known for the small 9 kDa subunit VHA-e in Arabidopsis. It consists of two membrane integral helices and a cytosolic C-terminal tail but the function of the subunit remains elusive. It may not be essential for V-ATPase activity in yeast and is absent in the vacuole in plants, but it might be required for V-ATPase assembly, since its absence results in a Vma^–^ phenotype in yeast (Sambade and Kane, 2004; Compton et al., 2006; Jaquinod et al., 2007; Seidel et al., 2008; Bueler and Rubinstein, 2015).

**FIGURE 4:**
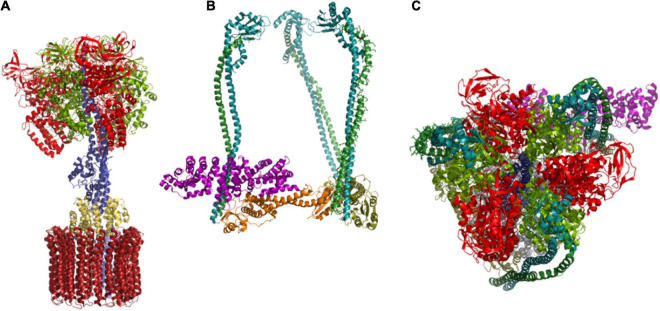
Structure-function relationship of the subunit arrangement. **(A)** Subunits that are directly involved in catalysis of proton pumping are given, starting with ATP-hydrolysis at the catalytic head, transformation of conformational changes into rotation of the central shaft by VHA-D and VHA-F, and VHA-d-mediated transduction of the rotation to the proteolipid ring, are given. VHA-a contributes the semi-channels for loading and unloading of proton binding sites. **(B)** The peripheral stalk subunits form a structure that resembles a rigid cage. It anchors the catalytic head to the membrane and prevents its co-rotation. Subunits of the peripheral stalk have a direct impact on coupling ratio. **(C)** Top view of the V_1_ sector reveals the arrangement of VHA-A subunits with VHA-D located in the center. The structures are based on pdb 3j9t (Zhao et al., 2015).

The hexamer of alternating subunits VHA-A and VHA-B form the core complex of the subsector V_1_ and resemble the catalytic head of F-ATP synthases (Grüber et al., 2001). Both subunits are capable of binding nucleotides, but only VHA-A maintains hydrolase activity during evolution and VHA-B takes over regulatory and structural functions *via* interactions with aldolase and the cytoskeleton (Chen et al., 2004; Lu et al., 2007). VHA-A consists of four domains, I–IV, with a nucleotide-binding P-loop in domains III and IV. Domain II has no counterpart in the F-ATP synthase subunit β and thus is called a non-homologous region (Shao et al., 2003; Maegawa et al., 2006). The first P-loop is responsible for ATP-hydrolysis and the target of redox modulation (Seidel et al., 2012). The hexameric head forms a central cavity, which is occupied by the central stalk, a heterodimer of subunits VHA-D and VHA-F, which are single copy genes in *A. thaliana*. These two transduce conformational alterations caused by the catalytic cycle of VHA-A into rotation while they are connected to the proteolipid ring *via* VHA-d. This arrangement demands a rigid structure to prevent co-rotation of the catalytic head (Figure 4). This structure is provided by a cage-like composition of three vertical peripheral stalks out of the elongated subunits VHA-E and VHA-G, which are crosslinked by the horizontally orientated VHA-C and VHA-H (Kitagawa et al., 2008; Zhao et al., 2015). Last but not least, interactions with VHA-a anchor the peripheral stalks to the membrane. The VHA-H and VHA-C from yeast have been the first V-ATPase subunits that were crystallized (Sagermann et al., 2001; Drory et al., 2004). The data revealed a bipartite structure of VHA-H with multiple domains for protein-protein interactions, which can even be divided into two independent peptides without loss of function (Liu et al., 2005), while VHA-C consists of a globular head and a foot domain connected by a bundle of α-helices. The N-terminal domain of VHA-H binds the VHA-E/VHA-G dimer with moderate affinity, and its interaction depends on ATP hydrolysis in yeast, whereas VHA-C binds to the heterodimer with high affinity (Sharma et al., 2018). Like VHA-B, VHA-C has been reported to interact with the actin cytoskeleton (Vitavska et al., 2005). VHA-B, VHA-E, and VHA-G are encoded by three genes in *A. thaliana* and ensure high flexibility in the formation of the V_1_-sector and, in particular, the peripheral stalk, and subunits VHA-A, VHA-C, VHA-D, VHA-F, and VHA-H are encoded by single-copy genes and might be the highly conserved and less flexible part of V_1_ (Sze et al., 2002). The expression of VHA-E isoforms is best analyzed. It is organ-specific and of developmental relevance in *A. thaliana*. During embryogenesis, VHA-E1 is the most important isoform, and VHA-E3 can be found in the endosperm (Strompen et al., 2005).

## Assembly of the V-ATPase Complex in the ER

Knowledge of the assembly of the entire complex is scarce, and although progress has been made in understanding the assembly of the V_0_-sector in yeast and mammals, there are only little data on the biosynthesis of plant V-ATPases. The situation becomes confusing because of the regulatory mechanism of reversible disassembly in yeast and mammals, which is often referred to as complex assembly in the literature. An early study on oat showed that V-ATPase is fully assembled already in the ER, so the entire biosynthesis of the complex takes place in the ER of plant cells where the assembly is assisted by the chaperones Calnexin and BiP (Herman et al., 1994; Li et al., 1998). This is supported by the observation that VHA-E locates first to the ER before its transport to the Golgi and finally to the vacuole, and that VHA-A interacts with the membrane before it interacts with VHA-B (Frey and Randall, 1998; Seidel et al., 2005). On the other hand, a concerted assembly pathway of V_0_ and V_1_ and a separate assembly of V_0_ and V_1_ with subsequent union in the Golgi were suggested as alternate assembly mechanisms in yeast in the past; all reported and suggested mechanisms might even co-exist (Kane, 1999).

The formation of the membrane integral sector requires a couple of additional assisting proteins like Vma12p, Vma21p, Vma22p, Voa1p, and the ribonuclease YPR170W_B in yeast. Vma21p interacts with VHA-c’ for proteolipid ring assembly, while a heterodimer of Vma12p and Vma22p facilitates the assembly of the proteolipid ring with VHA-a (Malkus et al., 2004). VHA-e also interacts with Vma21p, and it is suggested to complete the assembly (Compton et al., 2006). Next, the membrane integral α-helix of Voa1p interacts with VHA-c” and VHA-c and thereby forms a binding pocket for VHA-d (Ryan et al., 2008; Roh et al., 2018). The incorporation of VHA-d was thought to allow for the release from the ER and subsequent binding to the V_1_ sector in a sequential scenario (Graham et al., 1998; Ryan et al., 2008; Abbas et al., 2020).

Subcomplexes like VHA-A_2_/VHA-B_2_ and VHA-C_1_VHA-E_3_VHA-G_3_VHA-H_1_ were identified, which might represent intermediate states of assembly, and the same might be true for VHA-E/VHA-G, since VHA-E is unstable in the absence of VHA-G (Tomashek et al., 1997; Charsky et al., 2000; Fethiere et al., 2004; Zhang et al., 2008; Hildenbrand et al., 2010). These subcomplexes likely represent the building blocks for the assembly of V_1_-sectors. Their assembly with V_0_ is supported by the RAVE complex consisting of Rav1p, Rav2p, and Skp1p, and is best characterized as part of the re-assembly machinery and, thus, re-activation of the yeast V-ATPase after it dissociates into V_0_, V_1_, and VHA-C in the absence of glucose to downregulate ATP-consumption. V-ATPases seem to be even primed to disassemble in mammals (Parra and Hayek, 2018). However, the RAVE complex is also involved in biosynthesis of V-ATPases (Smardon and Kane, 2007). The complex is required to finally incorporate VHA-C into the complex and to ensure an appropriate orientation of V_1_ and V_0_ during V-ATPase (re-) assembly in yeast (Smardon et al., 2015). The recruitment of the RAVE-complex depends on intact V_0_-sectors where it binds Vph1p and not Stv1p exclusively in the presence of glucose (Smardon et al., 2014; Jaskolka and Kane, 2020). At the level of V_1_, RAVE undergoes a short sequence of interactions, binding first to V_1_ followed by VHA-C (Jaskolka et al., 2021). Abe et al. (2019) complemented yeast V-ATPase mutants with human subunits and suggested that lack of VHA-F or any V_0_ subunit results inVph1p being stalled in the ER and that lack of any V_1_ subunit results in cytosolic VHA-A. This observation indicates an efficient checkpoint that is located in the ER, presumably calnexin or BiP, so V-ATPases might be under the control of the canonical ER quality control and its calnexin/calreticulin cycle (Araki and Nagata, 2011).

Many other proteins have been reported as putative assembly factors in the past but have not been confirmed, such as PKR1 or VTC family proteins (Cohen et al., 1999; Davis-Kaplan et al., 2006). However, orthologous proteins of Vma12 and Vma21 have been identified in *A. thaliana*, and AtVma21a has been shown to interact transiently with VHA-c”, so VHA-c”, instead of VHA-c’, might recruit Vma21 for proteolipid ring assembly in plants (Neubert et al., 2008). This would compensate for the absence of VHA-c’ in higher eukaryotes. The subsequent transport is still a subject of investigation; on the one hand, VHA-E and VHA-a1 take the canonical route of the secretory pathway and Vma21 bears a dilysine motif for recycling back to the ER, on the other hand, VHA-a3-bearing V_0_-subunits have been observed to take a route to the vacuole bypassing the Golgi (Figure 1; Seidel et al., 2005, 2013; Neubert et al., 2008; Viotti et al., 2013; Lupanga et al., 2020).

## Enzymatic Properties and Enzyme Regulation

V-ATPase couples ATP hydrolysis to proton transport. V_1_ serves as ATPase with three catalytically active ATP-binding sites, one at each VHA-A. The activity of ATPase is in the range of 4.32 μmol ATP or released phosphate h^–1^ mg^–1^ protein to 18.7 μmol h^–1^ mg^–1^ (Dietz et al., 1998; Kawamura et al., 2000, 2001; Kłobus and Janicka-Russak, 2004; Chen et al., 2011). ATP-hydrolysis is well-coordinated and occurs in a defined and repeated sequence. This involves three different conformational states of VHA-A, which are dependent on the nucleotide binding state and drive the clockwise and stepwise rotations of the central stalk by VHA-D and VHA-F; the rotations create a torque of 36 ± 4 pN nm (Hirata et al., 2003). The central stalk transduces the rotations to the proteolipid ring, and then the proton binding sites are loaded by the cytoplasmic semi-channel of VHA-a. The ring makes an almost complete clockwise turn before proton binding interferes with the barrier charge, and the proton is released into the luminal semi-channel (Grabe et al., 2000). The coupling efficiency as proton to ATP ratio varies and depends on pH-difference ΔpH of the cytosol and the lumen. It is affected by an increasing proton shunt along the gradient with increasing ΔpH, and the ratio changes approximately by 0.7 protons per ATP per ΔpH (Kettner et al., 2003a; Rienmueller et al., 2012). In citrus, a high pH gradient and improved coupling are supported by the luminal counter ions malate and citrate (Müller and Taiz, 2002). In general, chloride acts as a counter-ion, and its uptake by anion channels and transporters like the endosomal AtCLC-d reduces the impact of the electrogenic component of the proton motive force and favors the proton gradient (Beyenbach and Wieczorek, 2006; Fecht-Bartenbach et al., 2007). The proteolipid ring and hemi-channels resemble a Brownian ratchet like the F_0_-sector of F-ATP synthases (Oster, 2002). Once the motor is stalled, wobbling of VHA-c proton binding sites between the hemi-channels of VHA-a facilitates proton slip flux together with high luminal proton concentrations (Grabe et al., 2000). A decrease in cytosolic pH further increases the affinity to ATP to counteract cytosolic acidification and the regulation pays attention to cytosolic and luminal pH; information on the pH might be transported to the other side of the membrane, possibly by protonation of proteins (Davies et al., 1994; Rienmueller et al., 2012). For completeness, ATP-synthesis was observed on the V-ATPase of maize vacuoles in the presence of a Me_2_SO-containing medium, which decreases the K_*m*_ for phosphate because of the altered solvation energy of phosphate, and this reaction was observed even in the absence of a proton gradient (Façanha and Okorokova-Façanha, 2008). The authors suggest that this might also occur as an energy-conserving mechanism in dehydrating environments like, for instance, seed cells. In yeast, the function of V-ATPase was reversed by applying an appropriate voltage (Kettner et al., 2003b).

Lipids might play a role in activating V-ATPase in their final destination. The phosphatidylinositol phosphate lipid composition of endomembrane compartments differs, whereas endosomes can be characterized by the presence of PI(3)P; the Golgi is enriched with PI(4)P, which interacts with the yeast VHA-a isoform Stv1p, while its vacuolar counterpart Vph1p interacts with PI(3,5)P_2_ in tonoplasts (Banerjee and Kane, 2020). PI(3,5)P_2_ might additionally contribute to luminal acidification in guard cells by blocking secondary active transport, and PI(3,5)P_2_ targets CLC-a and inhibits its anti-port activity and thus minimizes the consumption of proton motif force (Carpaneto et al., 2017; Scholz-Starke, 2017). This includes phosphatidyl 3-kinase, which interacts with VHA-B2 in the tonoplasts of Arabidopsis (Liu et al., 2016).

### Biochemical Regulation of V-ATPase

The activity of V-ATPase is modulated at the short-term level with respect to illumination of plants. This regulation is driven by cytoplasmic ATP/ADP-ratio, which is altered upon dark-light transitions, and blue light-driven phosphorylation of VHA-A and subsequent binding of 14-3-3 proteins. Light-dependent 14-3-3 protein binding might be one mechanism to coordinate the activity of proton pumps in the plasma membrane and endomembrane compartments (Dietz et al., 1998; Klychnikov et al., 2007; Cosse and Seidel, 2021). The main regulatory nucleotide binding sites might be located in VHA-C with its conformational alteration upon ADP-binding and its high affinity for ADP in yeast. This might also explain the diffuse stator arrangement of the V-ATPase from *Kalanchoe daigremontiana* in the absence of nucleotides; however, additional nucleotide binding sites of possible regulatory relevance can be found in VHA-B and VHA-A (Forgac, 1999; Domgall et al., 2002; Armbruster et al., 2005; Maegawa et al., 2006). An additional nucleotide-dependent mechanism is the blocking of anion channels of the CLC- and the ALMT-family by free ATP, which communicates the cellular energy status to the membranes and indirectly regulates V-ATPase activity (Angeli et al., 2016). In mammalian cells, the presence of cyclic AMP enables the trafficking of V-ATPases to the apical surface (McGuire et al., 2017).

Another key player in short-term regulation is calcium ions, which are linked to V-ATPases by calcineurin B-like (CBL) calcium sensors in plants. CBL2 and CBL3 can be found in tonoplasts, and as a target of CBL-interacting kinases CIPK9 and CIPK17, they are directly involved in V-ATPase activation and subsequent control of plant growth and ion homeostasis (Tang et al., 2012; Saito and Uozumi, 2020). A close relationship of calcium- and pH-homeostasis also results from the calcium transport and maintenance of resting calcium ion concentrations in the cytosol and is a general feature due to reports from yeast and protozoan and plants (Martinoia et al., 2007; Ma et al., 2021; Stasic et al., 2021). This interplay becomes obvious by the loss of the calcium proton exchanger Cax1 in *A. thaliana*, which results in 40% inhibition of V-ATPase (Cheng et al., 2003). Such coordination is achieved by calcium-dependent kinases independent of electrogenic effects on the membrane. The calcium-dependent kinase 1 (CDPK1) even activates the barley V-ATPase in response to gibberellic acid, thereby integrating hormonal and calcium-signaling in aleurone (McCubbin et al., 2004). To complete the information on regulation by phosphorylation, VHA-C bears multiple phosphorylation sites and is a target of the without a lysine kinase 8 (WNK8) (Hong-Hermesdorf et al., 2006).

**FIGURE 5:**
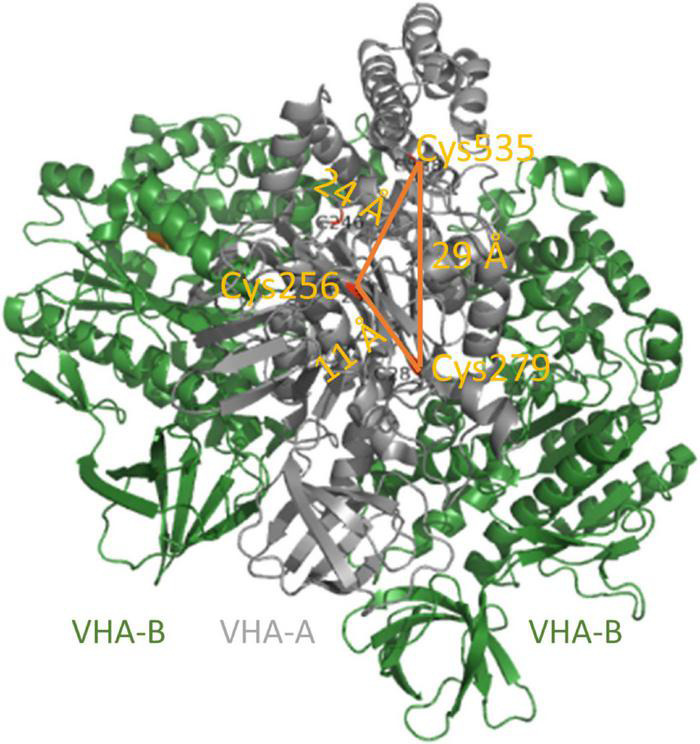
Conserved cysteine of VHA-A. VHA-A bears three conserved cysteines. Their distances of 11–29 Å exceed the maximum distance for disulfide formation. Cys256 is located in the catalytic ATP-binding P-loop, and its redox modulation efficiently inhibits V-ATPase activity. The structure is based on pdb 3j9t (Zhao et al., 2015).

Furthermore, V-ATPase is subject to redox modulation of cysteine residues as a highly conserved regulatory mechanism, and its catalytic activity decreases by addition of oxidants like hydrogen peroxide, nitric oxide, nitrate, oxidized glutathione (GSSG), and thioredoxin (TRX), or alkylating reagents like N-ethylmaleimide (NEM) and iodacetamide (IAA). Accordingly, reducing agents like glutathione, H_2_S, and DTT promote the activity (Hager and Biber, 1984; Hager and Lanz, 1989; Feng and Forgac, 1992a,b; Dschida and Bowman, 1995; Dietz et al., 1998; Tavakoli et al., 2001; Seidel et al., 2012; Kabala et al., 2019). Feng and Forgac suggested that the thiol switch in VHA-A is a redox-sensitive mechanism of the bovine V-ATPase: The active state is characterized by a disulfide bridge between cysteinyl residues Cys277 and Cys532, and the inactive state involves a disulfide bridge between Cys532 and Cys254. The latter cysteine residue resides in the ATP-binding P-loop of VHA-A, so disulfide bridge formation blocks the ATP-binding site (Feng and Forgac, 1992a,b, 1994). The bovine cysteine residues Cys254, Cys277, and Cys532 are highly conserved and correspond to Cys256, Cys279, and Cys535, respectively, in *A. thaliana* (Figure 5; Seidel et al., 2012). An inhibitory intramolecular disulfide formation within VHA-A has been ruled out for *A. thaliana* based on the data, that exclusively the replacement of Cys256 by a serine residue abolished the redox-sensitivity of V-ATPase activity (Kawamura et al., 2001; Tavakoli et al., 2001; Seidel, 2009; Seidel et al., 2012). The fact that a cysteine residue in an ATP-binding P-loop is common for many ATP-hydrolyzing enzymes (Yatsuhashi et al., 2002; Messens et al., 2004; Buhrman et al., 2005) and the finding that Cys256 is not conserved between V-ATPases and A-ATPases and is even replaced by serine in anaerobic and thermophilic Archaebacteria support the conclusion of redox-regulation exclusively *via* Cys256 (Maegawa et al., 2006). Still, the precise redox state of Cys256 in the inhibited state remains unclear. Possible posttranslational modification of cysteine residues comprises oxidation of thiolate anions to sulfenic acid, followed by oxidation to sulfinic and sulfonic acids, and glutathionylation, nitrosylation, and disulfide formation (Dietz, 2003; García-Santamarina et al., 2014; Fernando et al., 2019). Besides VHA-A, one cysteine (Cys179) is conserved in the VHA-B of all eukaryotes, and Cys134 and Cys186 are conserved in VHA-E of plants (Tavakoli et al., 2001). An intramolecular disulfide formation with a midpoint potential of -330 mV has been observed for VHA-E and linked to the nucleotide-binding state of VHA-A (Kawamura et al., 2001).

### Glucose-Dependent Regulation by Reversible Dissociation

**FIGURE 6:**
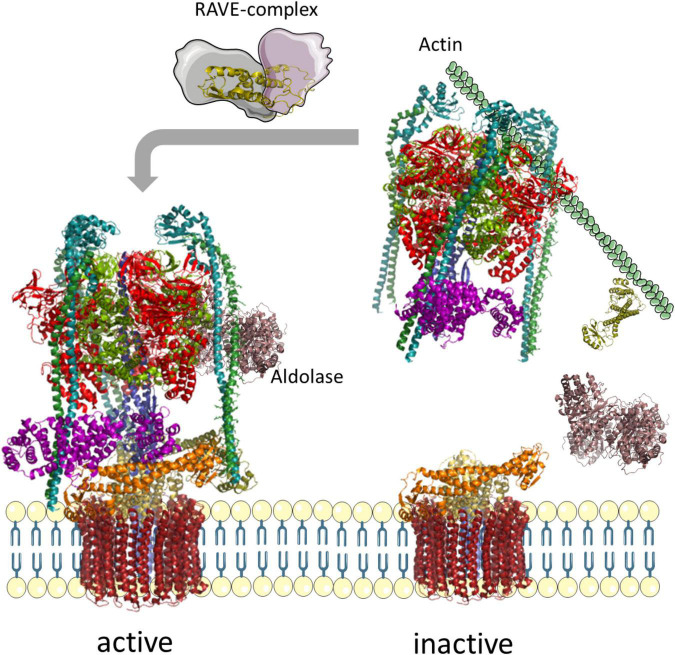
Reversible assembly of yeast V-ATPases. In the absence of glucose, the glycolytic aldolase dissociates from the complex, followed by VHA-C and the entire V_1_-sector; VHA-C and the residual V_1_ sector bind to actin to prevent free diffusion. Supply with glucose results in re-assembly of V-ATPase, mediated by the RAVE complex. The structure of V-ATPase relies on pdb-file 3j9t, the aldolase on 7ka2, and Skp1 on 5xyl (Zhao et al., 2015; Shukla et al., 2018; Cash et al., 2020).

The V-ATPase of mammalian cells and yeast has evolved a remarkable regulatory mechanism to inhibit V-ATPase when a cell runs out of glucose. Under this condition, the V_1_ sector detaches from the membrane, and ATP-hydrolysis and proton transport are inhibited (Figure 6). This is controlled by the glucose-sensitive signaling pathway of Ras-GTPases, Ira1p, Ira2p, and cAMP, and phosphorylation by protein kinase A (PKA) in yeast (Bond and Forgac, 2008). The level of glucose is also sensed by an interaction with a glycolytic aldolase, and the absence of glucose results in the release of aldolase and detachment of VHA-C and the residual V_1_ sector (Lu et al., 2001, 2004, 2007). In the disassembled state, the central pore is arrested by the N-terminal domain of VHA-a, which prevents rotation of the proteolipid ring and arrests the ring in a position with VHA-c” near transmembrane helices 7 and 8 of Vph1p, and thereby prevents passive transport of protons (Roh et al., 2018). Rotation of the central stalk and ATP hydrolysis are blocked by the C-terminal half of VHA-H, which interacts with the central stalk of VHA-D/-F (Sharma et al., 2018). Released V_1_ sectors and VHA-C both bind to the actin-cytoskeleton to maintain the complex close to the membrane and respective V_0_ complexes. Re-assembly is mediated by the rabconnectin-3 complexes of higher eukaryotes or the yeast RAVE complex. This complex consists of Rav1p, Rav2p, and Skp1p, which bind to V_1_ and VHA-C in a glucose-independent manner, but binding to the Vph1p of V_0_ is sensitive to glucose (Seol et al., 2001; Smardon et al., 2002, 2015; Smardon and Kane, 2007; Jaskolka and Kane, 2020). Actually, the RAVE complex brings V_0_, V_1_, and VHA-C in an appropriate position for re-assembly where the rotational state might become critical (Smardon et al., 2015; Zhao et al., 2015). The specificity of Vph1p restricts this regulation to V-ATPase in tonoplasts (Smardon et al., 2014). Phosphofructokinase 1 (PFK1) senses ATP availability, interacts with V-ATPase, and contributes in re-assembly (Chan and Parra, 2014; Chan et al., 2016). Together with binding of TORC1, this results in active V-ATPase supercomplexes (Deprez et al., 2018; Hayek et al., 2019). However, an investigation of the V_0_V_1_ assembly state in desoxyglucose-fed mesophyll cells of *A. thaliana* revealed a fully assembled complex, and only the conformation of VHA-C was altered. This alteration explains the tilted structure of Kalanchoe V-ATPase in the absence of ATP (Domgall et al., 2002; Schnitzer et al., 2011). Although these data point to the absence of reversible dissociation in plants, it should be considered that these were gathered with samples from autotrophic tissue, and that the situation might be different in heterotrophic plant cells. This would further explain why components of the RAVE complex can be found in Arabidopsis (Jaskolka and Kane, 2020).

## V-ATPase and Abiotic Stress Response: Some Examples

The expression of V-ATPase subunits varies with respect to environmental conditions. It depends on drought, cold stress, salinity, heavy metals, aluminum stress, osmotic stress, and oxidative stress, and shows ABA-dependency (Schulze et al., 2012; Yang et al., 2012; Vera-Estrella et al., 2017; Xu et al., 2017; Liu et al., 2018; Shi et al., 2018; Wu et al., 2019; Feng et al., 2020). Due to the multitude of genes encoding for V-ATPase subunits, regulation of the transcript level is rather complex; 26 genes encode for 13 subunits in *A. thaliana*, resulting in high number of possible combinations. Besides environmental influences, some isoforms show organ-specificity like VHA-E2 and VHA-G3, which are expressed in pollen, but most show stress-responsive expression patterns (Hanitzsch et al., 2007). In contrast to *A. thaliana* where VHA-A is encoded by a single gene and therefore vulnerable to mutations, it is encoded by at least two genes in rice, tomato, and carrot, which are organ-specific expressed (Gogarten et al., 1992; Bageshwar et al., 2005; Hanitzsch et al., 2007). At the level of the V_0_ sector, all five VHA-c isogenes were expressed in leaves, and the same was observed for VHA-c”1, while the transcript of VHA-c3 was additionally high in root caps of seedlings and the expression of VHA-c”2 was not detectable (Padmanaban et al., 2004; Hanitzsch et al., 2007; Seidel et al., 2008). Because of its role in plant stress response, V-ATPase has been described as an eco-enzyme. Best characterized is its importance under salinity and heavy metal stress, and many reports rely on transcript analysis of crops. Most plants deposit sodium ions in the vacuole, often within specialized cells or tissues like the epidermal bladder of the common ice plant. The transport of sodium ions is driven by sodium/proton exchangers (NHXs) and thus depends on the proton motif force (Zhou et al., 2011). Accordingly, salt-tolerant plants like *Lobularia maritima*, *Suaeda salsa*, *Mesembryanthemum crystallinum* (CAM state), and *Aeluropus lagopoides* showed at least higher expression and often higher activity of V-ATPases under salt stress (Tsiantis et al., 1996; Wang et al., 2001; Popova and Golldack, 2007; Ahmed et al., 2013; Cosentino et al., 2013; Sanadhya et al., 2015; Barkla et al., 2016). V-PPase and V-ATPase contribute differently in sodium sequestration, and uptake occurs mainly in the TGN and is driven by TGN-specific V-ATPases, for instance, in Arabidopsis and *M. crystallinum*, so V-PPase plays a minor role in sodium sequestration (Kabala and Klobus, 2001; Wang et al., 2001; Krebs et al., 2010; Yi et al., 2014; Barkla et al., 2016). Nevertheless, overexpression of V-PPases of other plant species like *S. salsa, Zoysia matrella* enhances the salt tolerance in *A. thaliana* (Guo et al., 2006; Chen et al., 2015), resulted in higher V-ATPase activity. Vice versa knock down of vha-A resulted in reduced V-PPase activity (Guo et al., 2006; Chen et al., 2015; Lv et al., 2017). The salinity-dependent activation of V-ATPases is organ-specific and drives root and shoot growth; subunits like VHA-c5 have been found to be expressed higher in root epidermal cells and the root elongation zone of *A. thaliana*; overexpression of VHA-B from wheat resulted in improved root elongation in *A. thaliana*, while shoot growth was observed for *S. salsa* (Yang et al., 2010; Wang et al., 2011; Zhou et al., 2018). V-ATPase activity is adjusted mostly by protein expression. Post translational modifications play a minor role under salinity, though some are known (Wang et al., 2001; Nasiri et al., 2012; Cosentino et al., 2013). Polyamines decrease proton pumping activity in cucumber, so salinity-dependent activation of proton pumps is accompanied by decrease in polyamines; this might occur by transamidation of proteins, since high expression levels of transglutamidases result in higher activity of V-PPase and V-ATPase (Janicka-Russak et al., 2010; Zhong et al., 2020). In apple, malate accumulates in the fruit under salt stress, mediated by the kinase SOS2L1, which interacts with VHA-B1, phosphorylates the subunit in Ser396, and results in shifting from malate metabolism to storage (Hu et al., 2016).

Heavy metal stress includes a broad range of responses to cadmium, copper, zinc, and nickel, and the role of V-ATPase has been analyzed excessively in many plants under heavy metal stress. The effect of heavy metals on vacuolar proton pumps has been different in cucumber than in other plants and does not involve redox modulation. Cadmium, zinc, and nickel have an inhibitory effect on V-ATPase and V-PPase, the latter is stimulated by low concentrations of 10 μM zinc or nickel, while copper stimulates V-ATPase activity, and pre-incubation with copper even stimulated both pumps. Thus, in cucumber, V-ATPase is not of importance under zinc and nickel stress, and the activity of the V-PPase is sufficient (Kabała et al., 2010, Kabala et al., 2013; Kabala and Janicka-Russak, 2011). In contrast to salinity and the situation in cucumber, the vacuolar uptake of zinc relies on the V-ATPase in tonoplasts in *A. thaliana* (Krebs et al., 2010). An inhibitory effect of zinc on V-ATPase has also been observed for *A. thaliana*, although here the protein level was decreased (Fukao et al., 2011). Finally, zinc results in alkanization of vacuoles and inhibits cell expansion *via* V-ATPase (Fukao and Ferjani, 2011). Alkanization of vacuoles due to the putative membrane-permeabilizing effect of cadmium, cobalt, and nickel is also known in yeast. Copper has a different effect on yeast vacuoles; it blocks SNARE pairing, inhibits proton pumping, and thereby prevents vacuolar fusion in yeast (Miner et al., 2019; Techo et al., 2020).

## V-ATPase and Biotic Stress Response: Target for Improved Pathogen Defense

The midgut of insects is easily accessible for insecticides. This accessibility also concerns V-ATPase, which controls the pH of the insect midgut and drives nutrient uptake (Zhuang et al., 1999; Harrison, 2001). Insecticides like destruxin B act on V-ATPase here (Muroi et al., 1994; Bandani et al., 2001). The disadvantage of such insecticides is their broad impact on all insects and, thus, their low specificity for pathogenic insects. Even toxins of higher species-specificity like tulipaline A, which affects the V-ATPase of nematodes, were also toxic for mammalian cells (Caboni et al., 2014), so they likely have unwanted side effects if applied in the field. Proteins of the knottin or cystine knot family like pea albumin 1b (PA1b) bind to VHA-c and VHA-e and result in selective inhibition of insect V-ATPases. PA1b represents the first peptidic inhibitor of V-ATPases (Chouabe et al., 2011; Muench et al., 2014), and other peptidic inhibitors are Cry-proteins from *Bacillus thuringiensis* that bind to VHA-B and actin and function as insecticidal agent (Chen et al., 2010; Shabbir et al., 2019). It turned out that the Asian corn borer *Ostrinia furnacalis* is able to develop resistance against Cry1 and reduces the efficiency of peptidic inhibitors (Shabbir et al., 2019). Later approaches to target the insect midgut V-ATPase applied RNAi, which is expressed as dsRNA by plant cells. This approach is highly specific for a single species and results in significant larval mortality. Most approaches addressed VHA-A, for instance, against the cotton bollworm, cotton mealy bug, tomato leaf miner, whiteflies, potato psyllid, and corn planthopper (Wuriyanghan and Falk, 2013; Yao et al., 2013; Thakur et al., 2014; Mao et al., 2015; Khan et al., 2018; Rahmani and Bandani, 2021). The efficiency as means of larval mortality can be further improved by addressing two genes of the herbivore insect and by lectins, which improve dsRNA delivery and increase the mortality of the army beetworm from 6–8 to 48% (Yao et al., 2013; Mao et al., 2015; Khan et al., 2018; Martinez et al., 2021).

## Transgenic Lines of Arabidopsis

The analysis of transgenic lines of *A. thaliana* shed light into the relevance of individual subunits and revealed the different functions of V-ATPases in the vacuole and the TGN (Table 1). The absence of V-ATPase in tonoplasts is achieved by double knockout of VHA-a2 and VHA-a3, resulting in reduced zinc tolerance and reduced vacuolar nitrate storage, which is compensated by higher nitrate assimilation. The transgenic plants revealed that in the vacuole V-PPase is sufficient for gametophyte and embryo development. They further showed reduced calcium content and leaf tip necrosis typical for mutants of vacuolar calcium/proton exchangers, accompanied by growth retardation (Krebs et al., 2010). The triple knockouts of VHA-a2, VHA-a3, and Fugu5, and VHA-a2, VHA-a3, and AVP1, which lack both proton pumps in tonoplasts, showed reduced vegetative growth of shoots and roots and produced few or even no seeds, respectively, but both triple knockouts were viable (Kriegel et al., 2015). The triple knockout of VHA-a2, VHA-a3, and AVP1 has further defects in the early stages of embryo development like altered vacuolar morphology and defective auxin transport by disturbed localization of the auxin transporter PIN1 (Jiang et al., 2020). Still, loss of VHA-a1 and, thus, loss of V-ATPases in the TGN result in higher salt sensitivity, cell wall defects, and impaired cell elongation, and link the defects to the TGN (Bruex et al., 2008; Krebs et al., 2010).

**TABLE 1:** Characterized lines.

Gene	Insertion	Mutagenesis	Expression	References
VHA-A	+	–		Dettmer et al., 2005
VHA-C, Det3	–	+		Poch et al., 1993
VHA-E1	–	+		Strompen et al., 2005
Vha-a2/VHA-a3	+	–		Krebs et al., 2010
VHA-a2/VHA-a3/AVP1	+	–		Kriegel et al., 2015
VHA-a2/VHA-a3/FUGU5	+	–		Kriegel et al., 2015
Vha-a2/VHA-a3	+	–	P_Ubi_-AVP1	Kriegel et al., 2015

Det3 has been the first characterized line with a mutation in a VHA-gene generated by 1,2:3,4-diepoxybutane mutagenesis of ecotype Columbia, which affected VHA-C in this case. It has been part of a screen for short hypocotyls of dark grown seedlings and has the phenotype of a light-grown plant. Accordingly, the mutation caused defects in cell elongation and stomatal closure, and reduced response to brassinosteroids; these are caused by pH increases in the TGN that affect the transport of cellulose synthase and brassinosteroid receptors (Poch et al., 1993; Schumacher et al., 1999; Allen et al., 2000; Luo et al., 2015). The peripheral stalk subunit VHA-E1 is encoded by the TUFF gene, and a mutant was generated by EMS mutagenesis in the *A. thaliana* ecotype Landsbergerecta (Ler), which is characterized by a mutation in a splice site of the gene. The mutation turned out to be embryo-lethal with drastic effects at the cellular level like altered Golgi organization and vacuolar morphology, defects in cell wall synthesis, and large cells with multiple nuclei (Strompen et al., 2005). Insertion into AT1G78900, which would result in a truncated form of VHA-A of 63 kDa instead of 68 kDa, causes male and partial female gametophyte lethality, if homozygous. At the cellular level, the effects resemble the observation on the TUFF mutant with its altered Golgi morphology (Dettmer et al., 2005).

### Fluorescent Protein Fusions of V-ATPase Subunits

Most transgenic lines have been complemented with fluorescent protein fusions, starting with Det3 in 1999 (Table 2). This line has been successfully complemented by GFP fusion of VHA-C and confirmed the presence of V-ATPase in the secretory pathway (Schumacher et al., 1999). Next was the insertion allele of VHA-A, which has been complemented by GFP fusion of VHA-A and has been the second transgenic line expressing a V-ATPase subunit fused to a fluorescent protein (Dettmer et al., 2005). Determination of VHA-a’s role in targeting the complex required the generation of fluorescently labeled VHA-a isoforms. Loss of VHA-a1 has been complemented by VHA-a1-GFP and VHA-a1-mRFP, VHA-a2 and VHA-a3 have been complemented with GFP and mRFP fusions, respectively (Dettmer et al., 2006; Bruex et al., 2008). VHA-a1-GFP and VHA-a3-mRFP expressing lines have been crossed to visualize both the TGN- and the tonoplast-V-ATPase. These combinations revealed the function of VHA-a in targeting V-ATPase (Viotti et al., 2013). A set of lines with chimeric proteins of the first 37, 85, 131, 179, and 228 amino acids of VHA-a1 and VHA-a3 revealed that the targeting domain (TD) resides between the amino acids L132 and E179 of VHA-a1; this region was placed in VHA-a3 (VHA-a3-a1-TD-GFP), and transgenic plants were generated for co-localization with VHA-a1-mRFP (Lupanga et al., 2020). Mutations and deletions were introduced into the TD and identified the importance of single amino acids for targeting the complex. Applying an inducible construct of dominant negative mutation of Sar1, the Rab-GTPase of COPII vesicles, fused to CFP, proved that the ER export of VHA-a1 is COPII-dependent but not the export of VHA-a3. The difference in transport has been discussed in the context of lacking glycosylation sites in VHA-a3 (Lupanga et al., 2020). Two lines were generated for co-localization of the V-ATPase assembly factors Vma12 and Vma21 with VHA-a3, but co-localization of VHA-a3 and assembly factors was not observed, pointing to a rapid and efficient ER export *via* direct contact sites of the ER and tonoplasts (Viotti et al., 2013). In yeast, Vma21 seems to be also involved in ER export and escorts the complex to COPII vesicles (Malkus et al., 2004). Unfortunately, transgenic lines that allow for co-localization of Vma21 and VHA-a1 in Arabidopsis are missing.

**TABLE 2:** Fluorescent protein reporter lines.

Construct 1	Construct 2	Construct 3	References
VHA-A-GFP		–	Dettmer et al., 2005
VHA-C-GFP		–	Schumacher et al., 1999
VHA-a1-GFP		–	Dettmer et al., 2006
VHA-a1-mRFP		–	Lupanga et al., 2020
VHA-a2-GFP		–	Dettmer et al., 2006
VHA-a3-GFP			Dettmer et al., 2006
VHA-a3-mRFP		–	Bruex et al., 2008
VHA-a1-GFP	VHA-a1-mRFP	–	Lupanga et al., 2020
VHA-a1-GFP	VHA-a3-mRFP	–	Viotti et al., 2013
VHA-a2-GFP	VHA-a3-mRFP	–	Bruex et al., 2008
VHA-a3-GFP	VMA12-mRFP	–	Viotti et al., 2013
Vma21-GFP	VHA-a3-mRFP	–	Viotti et al., 2013
VHA-a1NT37a3-GFP		–	Lupanga et al., 2020
VHA-a1NT85a3-GFP		–	Lupanga et al., 2020
VHA-a1NT131a3-GFP		–	Lupanga et al., 2020
VHA-a1NT179a3-GFP		–	Lupanga et al., 2020
VHA-a1NT228a3-GFP		–	Lupanga et al., 2020
VHA-a1-GFP (E161S)	VHA-a1-mRFP	–	Lupanga et al., 2020
VHA-a1-GFP (F134Y)	VHA-a1-mRFP	–	Lupanga et al., 2020
VHA-a1-GFP (L159T E161S)	VHA-a1-mRFP	–	Lupanga et al., 2020
VHA-a1-GFP (L159T)	VHA-a1-mRFP	–	Lupanga et al., 2020
VHA-a1-GFP (Δ EEI)	VHA-a1-mRFP	–	Lupanga et al., 2020
VHA-a1-GFP (ELE)	VHA-a1-mRFP	–	Lupanga et al., 2020
VHA-a1-GFP (E156Q)	VHA-a1-mRFP	–	Lupanga et al., 2020
VHA-a1-GFP (E156Q L159T)	VHA-a1-mRFP	–	Lupanga et al., 2020
VHA-a1 GFP	Sar1bGTP-CFP	–	Lupanga et al., 2020
VHA-a3 GFP	Sar1bGTP-CFP	–	Lupanga et al., 2020
VHA-a1 GFP	VHA-a3-mRFP	Sar1bGTP-CFP	Lupanga et al., 2020

### Available Transgenic Lines

The generation of knockout and knockdown lines is hampered by the observed (conditional) lethality in different developmental stages as summarized before. The high number of isoforms further results in high complexity and makes it difficult to silence a multi-copy subunit. In the following, the generation of transgenic lines is discusses based on available insertion lines and number of isogenes. According to The Arabidopsis Information Resource (TAIR, 24 March 2022; Lamesch et al., 2012)^1^ more than 2,304 transgenic lines exist with insertions or substitutions in VHA genes and the two Vma21 isoforms (Table 3 and Supplementary Table 1), and the highest contribution refers to VHA-c”2 with 1,275 lines. For subunits VHA-A, VHA-B1, VHA-C, VHA-e2, and the assembly factor Vma21b, there are no lines available that bear an insertion in an exon. VHA-A, VHA-C, VHA-D, VHA-F, and VHA-H are encoded by single copy genes, and knockout or knockdown lines have been already generated and characterized for VHA-A and VHA-C (refer to 7). Generation of homozygous knockdown or knockout lines of VHA-D, -F, and –H appears simple, since available lines (Supplementary Table 1) can be screened for homozygous lines but with high risk of lethality.

**TABLE 3:** Available insertion lines of *VHA* genes.

Subunit	AGI	Number of lines
VHA-A	AT1G87900	22
VHA-B1	AT1G76030	31
VHA-B2	AT4G38510	50
VHA-B3	AT1G20260	48
VHA-C	AT1G12840	59
VHA-D	AT3G58730	20
VHA-E1	AT4G11150	66
VHA-E2	AT3G08560	33
VHA-E3	AT1G64200	17
VHA-F	AT4G02260	13
VHA-G1	AT3G01390	28
VHA-G2	AT4G23710	69
VHA-G3	AT4G25950	14
VHA-H	AT3G42050	52
VHA-a1	AT2G28520	39
VHA-a2	AT2G21410	41
VHA-a3	AT4G39080	36
VHA-c1	AT4G34720	22
VHA-c2	AT1G19910	15
VHA-c3	AT4G38920	24
VHA-c4	AT1G75630	50
VHA-c5	AT2G16510	63
VHA-c”1	AT4G32530	38
VHA-c”2	AT2G25610	1,275
VHA-d1	AT3G28710	52
VHA-d2	AT3G28715	36
VHA-e1	AT5G55290	34
VHA-e2	AT4G26710	20
Vma21a	AT1G05780	28
Vma21b	AT2G31710	9

For the two-copy subunits VHA-c”, VHA-d, and VHA-e, crossing of two transgenic lines might work to generate a complete knockout of these subunits, and the similarity of the isoforms point to high redundancy. However, both VHA-d isoforms are adjacent genes on the same chromosome, making a required crossover very unlikely. For VHA-e2, most insertions are located in introns, and the efficiency of these are questionable. More promising is the crossing of transgenic lines of VHA-c”1 and VHA-c”2 that are located on different chromosomes.

Three copies are present in the genome of *A. thaliana* for VHA-a, VHA-E, and VHA-G with a good characterized division of labor for the organelle-specific VHA-a, the organ-specific VHA-E, and VHA-G isoforms (Strompen et al., 2005; Dettmer et al., 2006, 2010; Krebs et al., 2010). According to the localization of VHA-a isogenes on chromosomes 2 (VHA-a1 and VHA-a2) and 4 (VHA-a3), insertion lines have been crossed and new lines have been generated for the combinations VHA-a1/VHA-a3 and VHA-a2/VHA-a3 (Krebs et al., 2010; Viotti et al., 2013). These appear less redundant, and transgenic lines with effects on single isoform are powerful tools.

The situation is totally different for the five VHA-c isoforms where five genes encode for three different proteins in *A. thaliana*. VHA-c1, VHA-c3, and VHA-c5 are identical proteins, but at least VHA-c1 and VHA-c3 are expressed in different tissues. This might indicate that VHA-c isoforms are less redundant than one would expect, so the knockout of single isoforms can contribute to the understanding of V-ATPase isoenzymes in *A. thaliana*. Anyway, addressing all proteolipids requires techniques like CRISPR/Cas; the same is likely true for genes like the two encoding for VHA-d isoforms where crossing of available transgenic lines is not promising to gain a double knockout line.

## Conclusion

While the structure and rotational catalytic cycle of V-ATPase are now widely understood, knowledge of the assembly of the complex is still scarce and has not been the focus in recent years. Investigation of V-ATPase assembly and ER export remains one of the main future perspectives. Progress has been made on the level of regulation, and activating and inhibiting proteins have been identified and are now promising targets for engineering and modulating the catalytic activity. This approach appears superior to genetic modifications or overexpression of single V-ATPase subunits because of multiple isogenes and the resulting complexity and flexibility in complex formation. However, a set of VHA isogenes can, nowadays, be addressed in one step by gene editing techniques like CRISPR/Cas. This offers a multitude of new perspectives in the investigation of V-ATPases, facilitates the generation of transgenic lines, and thus overcomes the lack of transgenic line collections in other plant species. The current knowledge of regulation and transport routes reveals that there are vast differences between plants and mammals (or yeast) despite the nearly identical structure. These differences might pay tribute to autotrophic metabolism and the multiple functions of vacuolar compartments in plants.

## Author Contributions

The author confirms being the sole contributor of this work and has approved it for publication.

## Conflict of Interest

The author declares that the research was conducted in the absence of any commercial or financial relationships that could be construed as a potential conflict of interest.

## Publisher’s Note

All claims expressed in this article are solely those of the authors and do not necessarily represent those of their affiliated organizations, or those of the publisher, the editors and the reviewers. Any product that may be evaluated in this article, or claim that may be made by its manufacturer, is not guaranteed or endorsed by the publisher.

## Footnotes

1
www.arabidopsis.org
